# Conspecific alarm calls, but not food-associated calls, elicit affect-based and object-based mental representations in a bonobo (*Pan paniscus*)

**DOI:** 10.1098/rsos.241901

**Published:** 2025-03-05

**Authors:** Nicole J. Lahiff, Zanna Clay, Amanda J. Epping, Jared P. Taglialatela, Simon W. Townsend, Katie E. Slocombe

**Affiliations:** ^1^ Department of Evolutionary Anthropology, University of Zurich, Zurich, Switzerland; ^2^ Centre for the Interdisciplinary Study of Language Evolution, University of Zurich, Zurich, Switzerland; ^3^ Department of Psychology, University of York, York, UK; ^4^ Department of Psychology, Durham University, Durham, UK; ^5^ Ape Cognition and Conservation Initiative, Des Moines, IA, USA; ^6^ Department of Ecology, Evolution and Organismal Biology, Kennesaw State University, Kennesaw, GA, USA; ^7^ Department of Psychology, University of Warwick, Warwick, UK

**Keywords:** vocal communication, receiver psychology, mental representation, affect, reference, bonobo

## Abstract

Non-human vocalizations carrying information regarding external events have been likened to referential words and are thus integral for exploring the origins of linguistic reference. Previous research suggests receivers decode this referential information and some studies have indicated that such calls can, like in humans, evoke mental representations of the referent in receivers. However, the nature of these representations remains ambiguous. Specifically, whether calls elicit affect-based representations (e.g. signaller fear after alarm calls) or object-based representations (e.g. threats encountered by signallers after alarm calls), or both, in listeners remains untested. To investigate this, we conducted a match-to-sample task with a language-competent bonobo (Kanzi) asking him to match playbacks of conspecific alarm and food-associated calls to lexigrams representing either affect-based (‘scare’, ‘surprise’) or object-based (‘snake’, ‘food’) content. Kanzi matched alarm calls to ‘scare’ and ‘snake’ lexigrams at above chance levels regardless of caller familiarity but did not match food-associated calls to either ‘surprise’ or ‘food’ targets. We propose environmental cues are required to interpret food-associated calls that occur across a variety of contexts. These findings suggest bonobo alarm calls evoke object- and affect-based representations for Kanzi, indicating the mechanisms underlying the perception of non-human vocalizations may be more similar to those in language than previously thought.

## 1. Background

The semantic nature of language is often considered to distinguish it from other non-human communication systems. Through the systematic mapping of meaning onto words or signs, human language has unbounded potential to refer to objects and events in the surrounding world. The origins of this ability have ignited intense debate, with comparative data from non-human animals representing a key line of evidence capable of shedding light on the evolution of linguistic reference. Seminal work with predator-specific alarm calls in vervet monkeys (*Cercopithecus aethiops*) was the first to suggest that non-human vocalizations also held referential qualities beyond their affective content [1,2]. Acoustically distinct calls were deemed ‘functionally referential’ if they were reliably produced to a specific referent, and receivers responded to the call as they would when directly perceiving the referent themselves. In the 40 years following these initial discoveries, many additional primate and non-primate species have been found to produce functionally referential calls, some with more levels of complexity [3,4]. For example, meerkat alarm calls have been shown to provide listeners with information on both predator type and urgency [5,6], analogous to the dual encoding of affective and referential information in human words [7].

How similar the mechanisms underlying the production and perception of non-human functionally referential calls are to those underlying linguistic references is a question of enduring interest, and one that has caused a number of scholars to question the relevance of functionally referential calls for understanding the evolution of human reference [8,9]. From a production perspective, human referential communication is intentional; we purposefully communicate about objects or events, and this is recognized by others as an intentional act [10–12]. In contrast, the mechanisms underpinning functionally referential communication in animals are not specified in the definition of the term and remain untested in most species. Several influential scholars have argued that functionally referential calls are likely the product of arousal processes elicited by the external object or event they denote [13]; however, chimpanzee alarm and food-associated calls have now been shown to be produced voluntarily in a flexible goal-directed manner to change the behaviour of others [14,15]. These findings indicate, contrary to long-held assumptions, that chimpanzees are capable of intentional production of functionally referential calls, albeit with potentially different mentalistic processes underpinning this production compared to human communicative acts [7,16].

In terms of perception of functionally referential calls, it has been suggested that, unlike humans, receivers may be responding to differences in the acoustic features of calls [2,17,18], bypassing the need for receivers to cognitively appraise any informational content. To disentangle these possibilities, Zuberbühler *et al.* [19] conducted a prime–probe playback experiment with wild Diana monkeys (*Cercopithecus diana*). Within a trial, receivers were first exposed to a prime stimulus that varied across trials (e.g. playback of predator vocalizations or male alarm calls to predators), then 5 min later, the probe stimulus which was always a playback of predator vocalizations (e.g. leopard growls). Call receivers had diminished vocal responses to the probe stimulus when the referential content of the stimuli remained the same, regardless of the acoustic similarity of the prime and probe stimuli. In other words, monkeys responded similarly when primed with leopard growls (acoustically similar to leopard growl probe stimulus) or male leopard alarm calls (acoustically different from leopard growl probe stimulus). Conversely, strong alarm responses were given to the probe when both the referential content and acoustic structure differed from the prime (e.g. a male eagle alarm call followed by leopard growls). The authors argued that these results indicate listener responses are mediated by mental representations and these findings, along with others like it (see also [20–22]) show that receivers are attending to referential information encoded within vocalizations, rather than responding in an automatic way to the acoustic properties of calls.

However, the precise type of information listeners extract from functionally referential calls and the nature of any mental representations elicited are unclear. When listening vervet monkeys hear a leopard alarm call, if they form a mental representation, is the representation of the fear level of the caller (affect-based), or of a leopard (object-based), or both [7]? Although sometimes seen as a dichotomy, signals can contain varying amounts of both affective and referential information [23,24]. However, the degree to which non-human listeners are sensitive to these types of information and the degree to which they drive adaptive responses is largely unknown. Inferring the affective state of the speaker is argued to be highly automatic in humans [25–27] and has traditionally been assumed to be the mechanism by which non-human species process vocalizations; as emotional read-outs without the need for mental state attribution [9,28,29]. In contrast, retrieving the word–referent pairing and thus the meaning of a referential word in humans is cognitively more complex but has rarely been examined in non-humans.

There is some evidence in avian species, namely Japanese tits and coal tits, that recipients form mental representations of a predator upon hearing a functionally referential alarm call. Wild tits exposed to snake-specific ‘jar’ alarm calls, general ‘chicka’ alarm calls or recruitment calls only increase their scanning and approach behaviour to snake-like objects (moving sticks on the ground/alongside the tree trunk) following the snake-specific calls (conspecifics, Japanese tits [30]; heterospecifics, coal tits [31]). Furthermore, if the object was not snake-like (swinging sticks), the tits did not show the same predator-avoidance tactics. This suggests that the jar calls of Japanese tits generate an object-based mental representation of the threat which enables appropriate adaptive responses by the receivers.

While these findings are promising, there is a notable paucity of data from our closest-living relatives with whom we shared a most recent common ancestor [32]. Such data are particularly important for reconstructing the evolutionary roots of human referential abilities, namely whether representational mechanisms emerged from shared ancestry, or are derived traits in the human lineage, with non-human primate functional reference perception bearing a more superficial resemblance to human referential perception. To our knowledge, only one study has examined the mental representations elicited by vocalizations in great apes. Using an eye-tracking paradigm, Sato *et al*. [33] found that chimpanzees showed a gaze bias towards videos of snakes during playbacks of conspecific alarm calls, but not for food-associated calls or silence. Conversely, no gaze bias was found towards images of food during playbacks of food-associated vocalizations. Furthermore, the gaze bias towards snake videos was not present for scream vocalizations, suggesting that subjects were not simply associating vocalizations where signallers experience high arousal (i.e. alarm calls or screams) with the snake stimuli. However, as acknowledged by the authors, the interpretability of these findings is weakened by methodological issues such as pseudoreplication [34,35] since combinations of auditory and visual stimuli were repeated across multiple trials. Also, critically, the eye-tracking measurements were simultaneous to the exposure of the calls. Thus, a vocalization may prime attention to the most ecologically relevant stimuli, but it remains unclear whether such an association would be made without a visible and relevant referent present, and thus this paradigm may not probe the nature of the mental representations that the vocalizations promote. Other species, including humans and Japanese macaques [36,37], show preferential attention to images of snakes but this implicit and automatic detection is a different level from processing to explicit identification and labelling of threats [38,39]. Further investigation is therefore required to unpack the types of mental representations that calls evoke in an ape species.

To bridge this gap in our knowledge, we investigated what type of mental representation is elicited in one of our closest-living relatives, bonobos (*Pan paniscus*), when perceiving conspecific calls, specifically alarm calls and food-associated calls. In contrast to many species, where context-specific production of calls has been found but no experimental call perception data are available to test whether they qualify as functionally referential calls [40–43], curiously the converse is true in bonobos. While no work as yet has examined the context specificity of alarm and food-associated calls, experimental work has shown that listeners extract information about the presence of a threat from bonobo alarm calls, as it has been found that fewer individuals show startle responses when perceiving a viper model after hearing conspecific alarm vocalizations than individuals that received no vocal information, i.e. those that first discovered the snake model [44]. Regarding food-associated calls, playback experiments in captivity have demonstrated that sequences of these calls hold some referential content. Receivers extracted information about the quality of food discovered by the caller that informed their search strategies for food sources [45]. Whether adaptive conspecific responses to bonobo alarm calls and food-associated calls are driven by object-based representations or affect-based representations is, however, currently unknown. Specifically, whether alarm calls elicit mental representations of the threat itself (e.g. a snake) or the fearfulness of the caller, and whether food-associated calls elicit representations of food or the excitement of the caller, has yet to be examined. To address this issue, we conducted playback experiments within a match-to-sample (MTS) task with a language-competent bonobo, named Kanzi.

For four decades, Kanzi has been exposed to visuo-graphic symbols known as lexigrams, acquiring lexigram meaning through using lexigrams in interactions with human caregivers rather than formal conditioning-based training [46]. Kanzi has constant access to visual lexigram boards that he uses daily, which include lexigrams representing a broad range of vocabulary but importantly, include both objects (e.g. banana) and more abstract notions (e.g. ‘good’). Kanzi regularly completes MTS tasks that demonstrate his proficiency at matching lexigrams to their corresponding spoken word or photographic image [46,47]. Importantly, Kanzi’s ability to match spoken words to photos and arbitrary lexigrams provides strong evidence that communicative signals elicit mental representations in this non-human primate. Kanzi is unique among captive apes in terms of the constellation of abilities and experiences he possesses relevant to uncovering the presence and nature of the mental representations elicited by conspecific calls. Kanzi is necessarily therefore the only participant in the current study, which is designed to test the capacity of his species to form different types of mental representations from conspecific calls.

We capitalized on Kanzi’s knowledge of both affect-based (scare, surprise) and object-based (snake, food) lexigrams and his proficiency with MTS paradigms to directly probe whether conspecific vocalizations elicit affect-based or object-based referential representations, bypassing the need to rely on passive behavioural responses previously used to draw inferences regarding animal mental representations. We presented Kanzi with conspecific alarm and food-associated vocalizations recorded from both familiar groupmates and unfamiliar wild bonobos within a MTS task. After hearing the call, Kanzi was presented with three lexigrams, one of which matched the presumed meaning of the call. The matching lexigram was either affect-based (alarm calls: scare; food-associated calls: surprise) or object-based (alarm calls: snake; food-associated calls: food). While bonobo alarm calls are elicited by a variety of external dangers, we presented the snake lexigram as an example of a salient threat that alarm calls are produced in response to. If conspecific calls elicited affect-based representations, we expected Kanzi to match alarm and food-associated calls to the lexigrams ‘scare’ and ‘surprise’, respectively, at levels significantly above chance (here: 33%). If calls elicited an object-based representation, then we expected Kanzi to match alarm and food-associated calls to the lexigrams ‘snake’ and ‘food’, respectively, at levels significantly above chance. If the calls prompted both types of mental representation, we expected him to match calls significantly above chance levels to both ‘scare’ and ‘snake’ in alarm call trials, and ‘surprise’ and ‘food’ in food-associated call trials. Conversely, if conspecific calls did not evoke either type of representation, we expected Kanzi’s choice of a matching lexigram to occur at chance levels. Finally, as Kanzi was exposed to calls of both familiar and unfamiliar conspecifics, we expected him to either perform similarly or show higher accuracy at matching these lexigrams to calls from group members rather than unfamiliar individuals.

## 2. Study 1

### 2.1. Material and methods

Experiments were conducted at the Ape Cognition and Conservation Initiative (ACCI) in Iowa, USA. Data were collected across three time periods: July–August 2017, October–December 2017 and July–October 2019. Kanzi was tested using a touchscreen in his home enclosure while briefly separated (less than 60 minutes) from other group members. We used a MTS paradigm that Kanzi had extensive experience with from previous research [47]. In a MTS trial, Kanzi was presented with a sample (spoken word or a conspecific call or a photographic image), which was immediately followed by the presentation of three lexigram choices on a touchscreen monitor. One choice was the corresponding ‘match’ item to the sample (hereafter referred to as the target; e.g. if Kanzi was presented with the spoken word ‘apple’ the matching target was the lexigram for ‘apple’); the other two choices were ‘foil’ items which were unrelated to the sample, and therefore incorrect options. The duration between the appearance of the lexigram response options (one target, two foils) and Kanzi selecting a choice was recorded for all trials.

#### 2.1.1. The selection of lexigrams

Before testing Kanzi on the MTS task, we endeavoured to check that Kanzi had good comprehension of the lexigrams used in the task. Kanzi engaged daily with lexigrams during interactions with his caretakers, and therefore it is not possible to determine exactly how often he used each item or heard the word corresponding to each lexigram. Therefore, to select suitable foil lexigrams, we examined data collected in the 18−26 months before our study started by Rabinowitz [47] that detailed Kanzi’s performance investigating his accuracy matching regular spoken English noun words to lexigrams. We identified lexigrams on which he scored 75%+ accuracy (see electronic supplementary material for details of Rabinowitz [47] MTS sessions). From this set of 75%+ accuracy lexigrams, we chose physical items (e.g. balloon, car, key) that were not relevant to the tested call types, i.e. they were not edible or things that Kanzi found frightening. We also wanted to include action words (chase, groom, tickle) that were ‘bonobo-relevant’ lexigrams to ensure that Kanzi could not rely on selecting the only bonobo-relevant choice when hearing a playback of a conspecific vocalization. These were not all examined by Rabinowitz; thus his familiarity with these items was confirmed alongside our target lexigrams (method detailed below). His accuracy with the three ‘bonobo-relevant’ lexigrams ranged from 83 to 100% prior to the first testing session, indicating high familiarity.

For the target lexigrams, we chose ‘scare’ and ‘surprise’ to test affect-based representations and ‘snake’ and ‘food’ to test object-based representations. ‘Scare’ was used to communicate about fear or to label items as scary. ‘Surprise’ was used to communicate about happiness and unexpected positive events (favoured activities, or high-value enrichment). While none of these lexigrams were novel to Kanzi they were used relatively infrequently in daily interactions, so we sought to provide training on these four items. Kanzi was first required to match spoken English versions of these words to their corresponding lexigrams. In word–lexigram training sessions, trials for target and bonobo-relevant foil lexigrams were interspersed with filler trials using items that Rabinowitz [47] indicated he was 75%+ accurate on. Target items appeared every 4−6 trials. As our study spanned several periods of testing Kanzi was given multiple training sessions to refamiliarize him with these items prior to the testing of each target (see electronic supplementary material for details), but for each target lexigram, we considered his performance on the last two training sessions that included the target lexigram prior to the test session where the target lexigram first appeared. Kanzi scored 4/4 (100%) with ‘scare’ and 8/10 (80%) with ‘snake’, 10/10 (100%) with ‘surprise’ and 6/6 (100%) with ‘food’. Within these training trials, Kanzi’s mean accuracy with filler words was 78%, indicating that Kanzi was performing to a high standard with target and foil words immediately before testing.

Second, to ensure Kanzi was correctly associating lexigrams with real-world depictions of our object-based targets (snake, food) and not only the spoken English word we asked Kanzi to match photographs to their corresponding lexigrams. Ten unique photographs of (i) snakes and (ii) mixed types of foods were used as samples to be matched to lexigrams of snake and food, respectively. For the two sessions that included the target lexigram immediately prior to the first trial where the target lexigram was tested, Kanzi scored 9/10 (90%) with ‘snake’ and 18/20 (90%) with ‘food’, which was above his mean accuracy on filler trials (80%). As the affect-based targets (scare, surprise) had no one-to-one mapping to specific objects, it was not possible to test his familiarity with pictorial depictions of these lexigrams.

#### 2.1.2. Playback stimuli

Kanzi was presented with audio recordings of calls produced by familiar groupmates at ACCI, collected by N.J.L. and A.J.E., and unfamiliar conspecifics from a wild population in Lui Kotale, DRC, collected by Z.C. We therefore had four different categories of playback stimuli: (i) familiar alarm calls, (ii) unfamiliar alarm calls, (iii) familiar food calls, and (iv) unfamiliar food calls. Vocalizations from familiar and unfamiliar individuals were recorded using a Sennheiser K6 directional microphone and Marantz PMD661 or PMD660 solid state recorder. Food-associated calls were recorded when individuals were discovering or consuming a variety of food types. Alarm calls were elicited by a variety of external dangers or threats. At ACCI alarm calls were recorded during exposure to a model snake, loud noises such as local firework displays during holiday events or during thunderstorms and at Lui Kotale alarm calls were elicited from events such as a snake discovery and planes flying overhead. As the specificity of alarm calls has not been tested in bonobos, we hoped that Kanzi would be able to match alarm calls elicited by a variety of external threats to an example of a specific threat (snake) for which he possessed a lexigram.

Recordings from unfamiliar bonobos were cut into single calls using Praat acoustic analysis software (v 6.0.16) and optimized for voice analysis with a Gaussian window (analysis window length 0.05 s, 250 frequency steps, dynamic range 70 dB, 10 kHz frequency scale with a spectrogram view window 0−10 kHz). Raven (v 1.2) software was used to cut the familiar audio recordings into single calls and to fade background noise in and out for all stimuli before and after the call to avoid an abrupt onset and offset. Only calls with minimal background noise were used. The stimuli were approximately equal in duration (1 s), and Raven was used to equalize both familiar and unfamiliar calls in terms of root mean square (RMS) amplitude. We used an Anchor Mini-Vox speaker to play stimuli during testing. In total, we had 96 playback stimuli: 24 single calls for each of the four categories of stimuli; exemplars are available in the electronic supplementary material.

Finally, to avoid Kanzi reacting strongly to playbacks during test trials and consequently failing to respond, we created 18 additional stimuli to habituate Kanzi to hearing conspecific vocalizations during MTS sessions. Kanzi completed 1 session (*n* = 12 calls; 6 alarm calls, 6 food-associated calls; 3 familiar calls and 3 unfamiliar calls per call type) 3.5 months before testing. Kanzi then completed 3 further sessions 1 week prior to testing (*n* = 6 calls; 3 alarm calls, 3 food-associated calls; 2 familiar and 1 unfamiliar for each call type). These later sessions included 36 playback mock trials (each stimulus was presented twice per session) interspersed among filler trials. During all habituation trials, the matching lexigram was ‘food’ or ‘scare’. Kanzi was not rewarded on these mock trials, and they were excluded from our analyses; a report of his responses at this stage can be found in the electronic supplementary material. None of the 18 habituation vocalizations were used as stimuli in the main experiment.

Within the main experiment, Kanzi was exposed to each audio stimulus on two separate trials (see below), but he was also exposed to the same stimuli in other related experiments not reported here. Although he heard the calls outside of this study, there was a minimum of 6 months prior to testing where Kanzi had not been exposed to the stimuli and he was equally exposed to all stimuli.

#### 2.1.3. Categorization of call types

For food-associated vocalizations, several distinct call types have been documented for bonobos [48], namely barks, peeps, peep-yelps, yelps and grunts. Therefore, all food-associated vocal stimuli were categorized by call type to allow us to check whether Kanzi’s performance varied in accordance with call type. All stimuli were categorized by Z.C., who has extensive training and experience categorizing bonobo vocalizations, and were first screened by ear and then verified by examination of spectrograms [45,48]. All five food-associated call types were present in our vocal stimuli, as well as a graded yelp/grunt call. As stimuli were not initially selected according to the type of call, the stimulus set contained between 3 and 16 calls of each of these 6 identified types (see electronic supplementary material, table S5).

#### 2.1.4. Creation of match-to-sample trial stimuli

To create a MTS trial we had to pair the target lexigram with two foil lexigrams. In total, we had 24 unique foil pairs for test trials, which were randomly allocated to the 24 playback sounds in each of our 4 conditions (familiar alarm call; unfamiliar alarm call; familiar food-associated call; unfamiliar food-associated call). In total, 9/24 (38%) foil pairs contained one bonobo-relevant lexigram.

**Figure 1.**
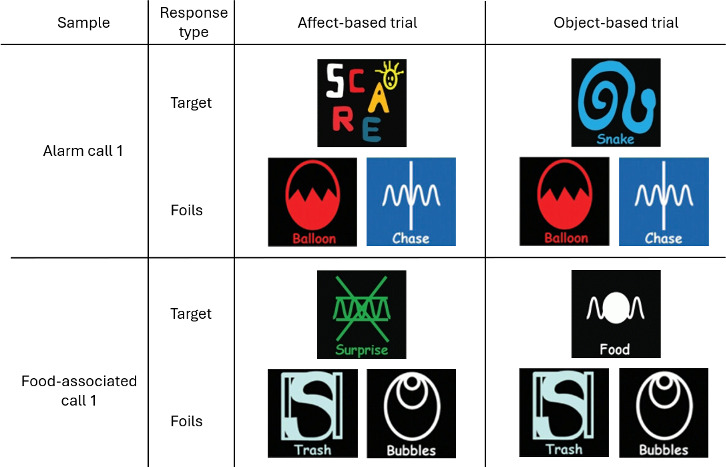
Example of three lexigram choices presented to Kanzi following a playback of a conspecific call. Target lexigrams remained consistent depending on the trial type (alarm call or food-associated call, affect-based or object-based response), but foil pairs changed between test trials, with 24 unique foil pairs presented for each playback sound condition (familiar alarm call, unfamiliar alarm call, familiar food-associated call, unfamiliar food-associated call) and presented once per target.

We formed two trials for each of the unique bonobo playback sounds (*N* = 96)—each exemplar was presented once with the relevant affect-based lexigram and once with the relevant object-based lexigram, to form a total of 192 trials. We presented the same two foils on both presentations of each playback sound (e.g. sample: alarm call 1; foil lexigram 1: balloon; foil lexigram 2: chase; target lexigram: scare on trial 1; snake on trial 2; see figure 1).

#### 2.1.5. Testing procedure

An experimental session consisted of (i) test trials (conspecific vocalization as the sample) and (ii) filler trials (spoken English word as the sample). There were 3−5 filler trials between each test trial. To maintain motivation during testing, Kanzi was given a food reward on the filler trial prior to a test trial if he chose the correct match. If incorrect, he was rewarded on the next correct trial following the test trial. Kanzi was never rewarded or received any feedback on a test trial. Sessions were made to consist of approximately 60 trials but were terminated early if Kanzi appeared distracted (e.g. frequently moving or orienting away from the touchscreen or if judged as so by both the researcher and caretakers present) and were resumed from the stopping point in the next session. Kanzi completed 25−60 trials in a single session, and he completed a maximum of 2 sessions in a day.

### 2.2. Results

Kanzi remained engaged throughout testing as indicated by his performance on filler trials; across all testing sessions, his mean accuracy on these trials was 82%. Further description of his performance on these trials is provided in the electronic supplementary material.

All analyses for test trials were conducted in R (v 4.1.2 [49]; with the interface RStudio 4.3.2). We conducted binomial tests where we compared observed proportions of correct responses to the test proportion which reflected chance levels of selecting the target lexigram (0.33, two-tailed). When presented with a conspecific alarm call, we found that Kanzi selected the ‘scare’ lexigram in 46/48 trials (0.96), a level significantly above than expected by chance (*p* < 0.001) and he selected the ‘snake’ lexigram in 29/48 trials (0.6), a level significantly above than expected by chance (*p* < 0.001). To explore whether he favoured the affect- or object-based response option, we conducted a Fisher’s exact test which revealed that Kanzi was significantly more likely to accurately match conspecific alarm calls to ‘scare’ than to ‘snake’ (*p* < 0.001).

In contrast, when presented with a conspecific food-associated call, Kanzi selected the ‘surprise’ lexigram in 7/48 trials (0.15), which was significantly less often than expected by chance (*p* = 0.005), and he selected the ‘food’ lexigram in 13/48 trials (0.27), which did not differ from chance (*p* = 0.445).

Kanzi’s average response times also reflected the pattern of the accuracy findings and were examined with two-tailed Mann–Whitney *U* tests. His median response time was significantly faster for alarm vocalizations (1.96 s) than food-associated vocalizations (2.30 s; *U* = 3181.5, *p* < 0.001), and within alarm calls, significantly faster for ‘scare’ (1.50 s) than ‘snake’ (2.26 s; *U* = 1720.5, *p* < 0.001; see electronic supplementary material for full description of response times).

We also examined whether there was a relationship between Kanzi’s performance on test trials and his familiarity with the caller, i.e. if his performance differed according to whether the playback call was the recording of a familiar group member or an unfamiliar wild bonobo. Overall, across both vocalization types (alarm, food-associated) Kanzi performed similarly with calls from familiar (45/96 correct) and unfamiliar individuals (50/96). To examine the effect of the familiarity of the caller for each call type (alarm, food-associated), we aimed to conduct four separate 2 × 2 contingency chi-square tests, one for each target lexigram (e.g. test 1 for lexigram ‘snake’ = familiar versus unfamiliar, number correct versus number incorrect). We first examined object-based representations and found there was no significant relationship between Kanzi’s accuracy and the familiarity of the caller for alarm calls (snake: *X*^2^ = 0.78, d.f. = 1, *p* = 0.376; table 1) but we did find a significant relationship between Kanzi’s performance and the familiarity of the caller for food-associated vocalizations (food: *X*^2^ = 5.17, d.f. = 1, *p* = 0.023; table 1), with Kanzi being more likely to be accurate with unfamiliar callers. With affect-based targets, the number of correct trials using the ‘scare’ response to familiar or unfamiliar callers was equal, so no further analysis was warranted (see table 1). For ‘surprise’, the expected frequencies for the chi-square tests were below 5; therefore, a Fisher’s exact test was conducted instead. We did not find a statistically significant association between caller familiarity and performance for the affect-based match ‘surprise’ (*p* = 1.000; table 1).

**Table 1.** Number of correct trials out of the total presentations for each target lexigram according to the familiarity of the caller (familiar, unfamiliar) with single call playbacks.

playback type	response to bonobo alarm vocalizations		response to bonobo food-associated vocalizations	
	‘scare’ target	‘snake’ target	‘surprise’ target	‘food’ target
familiar vocal samples	23/24	16/24	3/24	3/24
unfamiliar vocal samples	23/24	13/24	4/24	10/24

We did not identify any differences in Kanzi’s performance according to foil type; he performed similarly irrespective of whether test trials featured a bonobo-relevant foil or only noun word foils, but he did select a noun word foil rather than a bonobo-relevant foil in a larger percentage of incorrect test trials (see electronic supplementary material for further analysis on foil types).

Bonobos produce a range of different call types in response to food, and so for food-associated vocalizations, we also considered the possibility that only specific call types may transfer meaningful information about the context to listeners [50]. We examined the proportion of trials where Kanzi correctly selected ‘food’ or ‘surprise’ as a function of the type of call he heard. For ‘surprise’, Kanzi performed best with grunt calls (1/3, 33%) and worst with graded yelp/grunt calls (0/3, 0%). For ‘food’, Kanzi performed best with peep calls (6/14, 43%) and again had the lowest performance with graded yelp/grunt calls (0/3, 0%). Because our stimuli were not initially selected for call type, the sample sizes for some call types are small and therefore caution is warranted when considering how meaningful the descriptive differences in Kanzi’s performance were with different call types. See electronic supplementary material for a full description of Kanzi’s performance according to call type.

## 3. Study 2

Following Kanzi’s poor performance on food-associated vocalizations, we speculated that this may have been because single calls did not hold enough meaningful information for him to form affect- or object-based representations. Previous research with bonobos has indicated that sequences of food-associated calls influence foraging decisions [45], whereas the information content of single calls was not tested. Thus, the next step was to ascertain if Kanzi’s performance would change when exposed to food-associated vocalizations as call sequences. We asked Kanzi to first select lexigram responses (as in study 1), and then to select photograph responses. The latter was to ensure that his low performance was not due to any disconnect between his real-world encounters with food and the lexigram ‘food’. However, as this method required photographs, it was only appropriate for our object-based target ‘food’ and was not explored for the affect-based target ‘surprise’.

### 3.1. Material and methods

Data were collected from Kanzi at ACCI November–February 2018 and October 2019. We employed the same MTS paradigm as described in study 1; following the playback of food-associated call sequences, Kanzi was presented with three choices consisting of a target and two foil options. The affect-based target was ‘surprise’ and the object-based target was ‘food’. His response times for each trial were recorded.

#### 3.1.1. The selection of lexigrams

The target lexigrams were ‘surprise’ and ‘food’, and we included the same noun-word and bonobo-relevant foil choices as presented in study 1 for the vocalization-to-lexigram test trials. For vocalization-to-photograph trials, the target photographs were images of mixed fruits and vegetables. Here, we only included ‘groom’ and ‘tickle’ as bonobo-relevant foils since no photos adequately captured the formerly used ‘chase’.

Trials for study 2 took place alongside or just after study 1 sessions; therefore, no further training or refamiliarization was needed for study 2.

#### 3.1.2. Playback stimuli

The stimuli were from the same groups of individuals as study 1; group members at ACCI and individuals from Lui Kotale, DRC. We thus had two conditions here: (i) familiar food-associated call sequences and (ii) unfamiliar food-associated call sequences. We created 24 stimuli for each condition. We identified naturally occurring call sequences from our recordings and created three call sequences consisting of novel calls not presented in study 1 for 24/24 familiar playback stimuli and 22/24 unfamiliar playback stimuli. Two call sequences from unfamiliar individuals contained two novel calls and one call that was played in study 1. The same acoustic software and settings were used to create the stimuli as in study 1. Inter-call durations were standardized to 0.5 s to create stimuli consisting of three equally spaced calls with the total duration of the stimulus ranging from 2 to 5 s. Raven 1.2 was used to equalize the calls in terms of RMS amplitude. We used an Anchor Mini-Vox speaker to playback stimuli during testing. Exemplars of food-call sequence stimuli are available in the electronic supplementary material.

Kanzi was exposed to each audio stimuli three times during testing for this study: once for the affect-based target ‘surprise’, once for the object-based target ‘food’, and once to test the target ‘food’ using photograph responses instead of lexigrams. There was a minimum of 7 weeks between the test sessions containing the same vocal stimulus.

#### 3.1.3. Categorization of call types

To check whether Kanzi’s performance varied depending on the calls present in a sequence, all stimuli were again categorized by Z.C. They were first screened by ear and then verified by examination of spectrograms. Since we created stimuli from natural call sequences, we did not control for what call types were present within the sequence. Sequences could therefore consist of only one call type or two different call types. There were 15 unique combinations of calls, with between 1 and 13 exemplars of each one (see electronic supplementary material, table S6).

#### 3.1.4. Creation of match-to-sample trial stimuli

We paired our target choices (in both lexigram and photograph format) with two foils. As in study 1, we had 24 unique foil pairs for test trials, which were randomly allocated to the 24 playback sounds in each of our 2 conditions (familiar food call; unfamiliar food call). In vocalization-to-lexigram trials, 9/24 (38%) foil pairs contained one bonobo-relevant lexigram. In the vocalization-to-photo MTS task, bonobo-relevant foils appeared in 6/24 foil pairs allocated to familiar calls and 7/24 foil pairs allocated to unfamiliar calls as the formerly used choice ‘chase’ had to be discarded.

In vocalization-to-lexigram trials, two trials were created for each of the unique bonobo playback sounds (*n* = 48)—each exemplar was presented once with the relevant affect-based lexigram and once with the relevant object-based lexigram, to form a total of 96 trials. Like the former study, we presented the same two foils on the two presentations of each playback sound (e.g. sample: food call 1; foil lexigram 1: groom; foil lexigram 2: balloon; matching lexigram: surprise on trial 1; food on trial 2). As only the object-based target ‘food’ was tested in the vocalization-to-photograph trials, only one trial was created for each of the bonobo playback sounds (*n* = 24). The photographs for the target ‘food’ comprised different images of mixed fruit and vegetables that Kanzi was familiar with.

#### 3.1.5. Testing procedure

All experimental sessions consisted of (i) test trials (conspecific vocalization as the sample) and (ii) filler trials (spoken English word as the sample). There were 3−5 filler trials between each test trial. To ensure Kanzi remained engaged during testing he was given a food reward on the filler trial prior to a test trial if he selected a correct match. If incorrect, he was rewarded on the next correct trial following the test trial. Like study 1, Kanzi was never rewarded or received any feedback on a test trial. Sessions were terminated early if Kanzi appeared distracted and were resumed from the stopping point in the next session. Kanzi completed 59−60 trials in a single session, and he completed a maximum of 2 sessions in a day.

### 3.2. Results

Kanzi remained motivated throughout testing; for vocalization-to-lexigram trials his mean accuracy on filler trials was 79%. For vocalization-to-photograph trials his mean accuracy on these trials was 80%. Further description of his performance on these trials is provided in the electronic supplementary material.

All analyses were conducted in R (v 4.1.2). Binomial tests were conducted to compare observed proportions of correct responses to the test proportions which reflected chance levels (0.33, two-tailed). We first examined his performance when he was required to select a lexigram as a response. When presented with a 3-call sequence of food-associated vocalizations, Kanzi selected the ‘surprise’ lexigram in 7/48 trials (0.15) which was significantly lower than expected by chance levels (*p* = 0.005), and he selected the ‘food’ lexigram in 15/48 trials (0.31) which did not differ from chance (*p* = 0.879). It was then tested whether Kanzi would improve on the object-based match ‘food’ if he could respond by selecting a photograph, rather than a lexigram. When presented with call sequences and responding with a photograph, Kanzi selected the ‘food’ image in 17/48 trials (0.35) which did not differ significantly from chance (*p* = 0.759).

We again investigated whether the familiarity of the caller (group member or wild bonobo) affected Kanzi’s performance on test trials. Table 2 shows the number of correct trials when Kanzi was presented with lexigram response options and photograph response options. A chi-square test for independence indicated that there was no significant relationship between Kanzi’s performance and the familiarity of the caller for the lexigram ‘food’ (*X*^2^ = 0.38, d.f. = 1, *p* = 0.540), or the photograph of ‘food’ (*X*^2^ = 0.09, d.f. = 1, *p* = 0.762). For the lexigram ‘surprise’ the expected frequencies for the chi square test were below five therefore a Fisher’s exact test was conducted and found no association between Kanzi’s performance and the familiarity of the caller (*p* = 0.666).

**Table 2.** Number of correct trials out of the total presentations for each target lexigram according to the familiarity of the caller (familiar, unfamiliar) with food-associated call sequence playbacks.

playback type	food-associated call sequences with lexigram response		food-associated call sequences with photograph response
	‘surprise’ target	‘food’ target	‘food’ target
familiar vocal samples	4/24	7/24	8/24
unfamiliar vocal samples	3/24	8/24	9/24

As in study 1, we did not identify any differences in Kanzi’s performance according to foil-type; he performed similarly irrespective of whether test trials featured a bonobo-relevant foil or only noun word foils (see electronic supplementary material for further analysis on foil types). When considering the effects of call type, for the target ‘surprise’ his performance was best when all calls in the sequence were graded yelp/grunts (1/1, 100%) or a mixture of peep-yelps and yelps (2/2, 100%). For most call sequences, regardless of call types included, Kanzi scored 0% with the target ‘surprise’. For ‘food’ Kanzi performed best with call sequences where all the calls were grunts (2/2, 100%), sequences that contained both peeps and grunts (1/1, 100%), and sequences that contained yelps and grunts (3/3, 100%). With this target, his lowest performances (0%) were when all three calls in the sequence were yelps or graded yelp/barks, or sequences that contained a mixture of barks and peeps, peeps and peep-whistles, peep-yelps and other, or yelps and other. However, the small sample sizes available for each type of call sequence mean that these results should be interpreted cautiously. See electronic supplementary material for a full description of Kanzi’s performance according to call type.

## 4. Discussion

Previous observational and experimental work has revealed that the acoustic structure of animal vocalizations can convey to listeners both referential information about external objects and events in the world and information regarding the signaller’s motivational state [6,51]. Furthermore, research shows that the referential component can also evoke a mental representation of the threat in receivers [30,31]. Here, we build on these findings and demonstrate, for the first time, that a non-human species can form mental representations both of the affective state of the signaller and of a physical external object from conspecific vocalizations. Kanzi’s ability to match alarm calls to lexigrams of ‘scare’ and ‘snake’ irrespective of his familiarity with the caller indicates the robust nature of the representations that these calls elicit. Contrary to the traditional view of animal vocalizations [9,28,29] both affect-based and object-based representations were elicited in Kanzi by conspecific alarm calls, suggesting that there may be more continuity in the way in which vocalizations are processed in human and non-human species than previously assumed [7,52].

Unexpectedly, despite the previous experimental work showing short sequences of bonobo food-associated calls provided listeners with information on food quality [45], Kanzi did not appear to form object-based or affect-based mental representations for these vocalizations. In study 2, we gave Kanzi the opportunity to match food call sequences, rather than single calls, to both lexigrams and photos, but Kanzi failed to perform above chance with any of these methodological variations, despite his success with alarm calls. It may be that the differences between our findings and those of Clay & Zuberbühler [45] result from the very different methodological approaches used. In the current work, Kanzi was in a highly controlled setting with an absence of other environmental cues available to aid his interpretation of vocalizations, whereas Clay & Zuberbühler [45] presented vocal playbacks in whole group foraging tasks. Replication of Clay & Zuberbühler’s [45] study in different populations of bonobos would help to understand whether Kanzi is alone in being unable to form the tested representations, or whether such variability is present in other populations too. However, potential parallels can be drawn between our pattern of findings and Sato *et al*.’s [33] eye-tracking study that found chimpanzees preferentially attended to photos of snakes while listening to alarm calls, but again failed to show an attentive preference for photos of food while hearing food-associated calls.

What might explain Kanzi’s failure to match food-associated calls to the object-based referent? One possibility may be that the formation of mental representations in bonobos depends upon the type of calls perceived. Previous research suggests that some food-associated call sequences have an ambiguous meaning (a low correlation to specific events which results in listeners foraging in incorrect locations [45]). We also detected some variation in Kanzi’s accuracy for matching food call stimuli to the target lexigrams according to call type. It is therefore possible that our stimuli included a mix of referential and non-referential calls produced within this context which prevented Kanzi from forming specific mental representations. Another possibility already alluded to is that to generate a mental representation regarding an object-based referent receivers require further contextual cues to be available. Although detailed acoustic analysis and focal animal vocal data are needed to test the context-specificity of bonobo food-associated calls, certain general call types produced in feeding contexts are also reported in other contexts (e.g. travel [50]). Hence, perhaps for these call types, pragmatics is crucial in helping receivers disambiguate the associated referential content. To illustrate, if receivers hear peeps being produced from the canopy then this contextual information can be integrated to deduce that these calls are likely *food-related*. Follow-up experiments directly comparing the call types and investigating the impact of contextual cues would be beneficial to uncover why food-associated calls, in contrast to alarm calls, did not elicit mental representations of object-based reference for Kanzi. Finally, although the specificity of bonobo food-associated calls is unknown, it is possible that food-associated calls generate highly specific mental representations of the food type being consumed (e.g. calls given when consuming bananas elicit a mental representation of bananas). If this is the case, then Kanzi may have struggled to match food-associated calls given to specific food types with the meta-category of ‘food’. Presenting Kanzi with stimuli given to single food types (e.g. grapes) with their matching lexigrams (e.g. grape) could allow useful exploration of this possibility.

When considering Kanzi’s failure to match food-associated calls to the affect-based referent, it is possible that the stimuli, although all recorded in feeding contexts, actually varied in their affective content. The target lexigram ‘surprise’ is exclusively used by Kanzi and his caregivers to communicate about positive affect and positive events, yet the affective state of the signaller when producing calls in a feeding context may not have been consistently positive. While the discovery of food is likely to be highly positive, competition for resources may elicit negative affect, particularly for males who have lower priority access in small feeding patches [53,54]. In the future, controlling for the rank and recent history of the individuals providing food-associated call stimuli may help rule out this alternative explanation.

In potential contrast to the food-associated calls, the emotional content of the alarm call vocalizations was potentially more stable across stimuli and hence more likely to consistently match the negative valence of the target lexigram ‘scare’. Conversely, the referential content of the alarm calls may not have been as consistent across trials. Although Kanzi was above chance at matching alarm calls to the object-based target ‘snake’, he was more accurate and faster at matching vocalizations to the affect-based target ‘scare’. Although ‘snake’ was a salient and appropriate object-based referent for alarm calls since the discovery of snakes reliably elicits alarm calls in both Kanzi’s captive group and the wild population, his diminished performance on these trials may be because our alarm call samples were not always recorded responses towards snakes. Instead, alarm vocalizations were elicited by a variety of frightening stimuli or events (only 1−18 of 48 alarm vocalizations were elicited by a snake (1 confirmed; 17 recordings from the wild where it was impossible to confirm the source of distress)) to create our stimuli. Unlike smaller-bodied species, bonobos do not face a wide array of different predatory threats requiring different adaptive responses; thus it seems unlikely that they have acoustically distinct calls for multiple different threats, but the degree of specificity in the bonobo alarm calling system has not been tested. It is therefore possible that alarm calls activated multiple competing object-based representations that Kanzi had to choose between, leading to a longer response time to select the choice ‘snake’ than the affect-based choice ‘scare’.

By working with a language-competent ape and using a MTS paradigm, we demonstrate that Kanzi could not only implicitly associate conspecific vocalizations to relevant external referents [33] but uniquely also explicitly label calls in relation to affective content and physical entities. Kanzi’s ability to use symbolic communication provided us with a unique window into the nature of the mental representations elicited by conspecific calls. Indeed, his exceptional capacity to communicate with lexigrams provided him with the means to label calls and thus allow for the externalization of mental representations. Future work using matching of calls to photos rather than lexigrams in primate and non-primate species without experience with human language will be critical in shedding light on the potential scaffolding role of symbol use in the ability to represent affect- and object-based referents.

Ape language studies have been met with accusations of overly rich interpretations and debates over what can be concluded from reports of language comprehension and the use of symbols to communicate [55,56]. However, language-competent apes provide invaluable insight into cognitive and communicative capacities that are immeasurable in other members of the species [57]. Despite this, these animal models are often neglected. Our findings may result from one individual, but they have demonstrated for the first time that a non-human animal has the capability to form and externalize mental representations of both a signaller’s affective state and an external referent. We show that Kanzi not only perceives acoustic differences in conspecific vocalizations, he also generates multiple mental representations of the informational content of calls, demonstrating one of the closest homologues to the perception of spoken language known thus far.

## Data Availability

Data are available as electronic supplementary material [58].

## References

[1] Struhsaker TT. 1967 Auditory communication among vervet monkeys (*Cercopithecus aetthiops*). In Social communication among primates, pp. 281–324. Chicago, IL: University of Chicago Press. (10.1163/156853967X00073)

[2] Seyfarth RM, Cheney DL, Marler P. 1980 Vervet monkey alarm calls: semantic communication in a free-ranging primate. Anim. Behav. **28**, 1070–1094. (10.1016/s0003-3472(80)80097-2)

[3] Gill SA, Bierema A ‐K. 2013 On the meaning of alarm calls: a review of functional reference in avian alarm calling. Ethology **119**, 449–461. (10.1111/eth.12097)

[4] Townsend SW, Manser MB. 2013 Functionally referential communication in mammals: the past, present and the future. Ethology **119**, 1–11. (10.1111/eth.12015)

[5] Manser MB. 2001 The acoustic structure of suricates’ alarm calls varies with predator type and the level of response urgency. Proc. R. Soc. B **268**, 2315–2324. (10.1098/rspb.2001.1773) PMC1088882 11703871

[6] Manser MB, Seyfarth RM, Cheney DL. 2002 Suricate alarm calls signal predator class and urgency. Trends Cogn. Sci. **6**, 55–57. (10.1016/s1364-6613(00)01840-4) 15866180

[7] Heesen R, Sievers C, Gruber T, Clay Z. 2022 Primate communication: affective, intentional, or both? In Primate cognitive studies (eds BL Schwartz, MJ Beran), pp. 411–438. Cambridge, UK: Cambridge University Press. (10.1017/9781108955836.017)

[8] Wheeler BC, Fischer J. 2012 Functionally referential signals: a promising paradigm whose time has passed. Evol. Anthropol. **21**, 195–205. (10.1002/evan.21319) 23074065

[9] Rendall D, Owren MJ, Ryan MJ. 2009 What do animal signals mean? Anim. Behav. **78**, 233–240. (10.1016/j.anbehav.2009.06.007)

[10] Sperber D, Wilson D. 1986 Relevance: communication and cognition. Cambridge, MA: Harvard University Press.

[11] Donnellan E, Bannard C, McGillion ML, Slocombe KE, Matthews D. 2020 Infants’ intentionally communicative vocalizations elicit responses from caregivers and are the best predictors of the transition to language: a longitudinal investigation of infants’ vocalizations, gestures and word production. Dev. Sci. **23**. (10.1111/desc.12843) 31045301

[12] Moore R. 2017 Gricean communication and cognitive development. Philos. Q. **67**, 303–326. (10.1093/pq/pqw049)

[13] Tomasello M. 2008 Origins of human communication. Cambridge, MA: MIT Press.

[14] Schel AM, Machanda Z, Townsend SW, Zuberbühler K, Slocombe KE. 2013 Chimpanzee food calls are directed at specific individuals. Anim. Behav. **86**, 955–965. (10.1016/j.anbehav.2013.08.013)

[15] Schel AM, Townsend SW, Machanda Z, Zuberbühler K, Slocombe KE. 2013 Chimpanzee alarm call production meets key criteria for intentionality. PLoS ONE **8**, e76674. (10.1371/journal.pone.0076674) 24146908 PMC3797826

[16] Warren E, Call J. 2022 Inferential communication: bridging the gap between intentional and ostensive communication in non-human primates. Front. Psychol. **12**, 718251. (10.3389/fpsyg.2021.718251) 35095633 PMC8795877

[17] Owings DH, Beecher MD, Thompson NS (eds). 1997 Perspectives in ethology, vol. 12: communication. New York, NY: Plenum Press.

[18] Cheney D, Seyfarth R. 1990 How monkeys see the world: inside the mind of another species. Chicago, IL: University of Chicago Press. (10.7208/chicago/9780226218526.001.0001)

[19] Zuberbühler K, Cheney DL, Seyfarth RM. 1999 Conceptual semantics in a nonhuman primate. J. Comp. Psychol. **113**, 33–42. (10.1037//0735-7036.113.1.33)

[20] Evans CS, Evans L. 2007 Representational signalling in birds. Biol. Lett. **3**, 8–11. (10.1098/rsbl.2006.0561) 17443952 PMC2373811

[21] Cheney D, Seyfarth R. 1990 Attending to behaviour versus attending to knowledge: examining monkeys’ attribution of mental states. Anim. Behav. **40**, 742–753. (10.1016/s0003-3472(05)80703-1)

[22] Zuberbühler K. 2000 Causal cognition in a non-human primate: field playback experiments with Diana monkeys. Cognition **76**, 195–207. (10.1016/s0010-0277(00)00079-2) 10913576

[23] Gouzoules H, Gouzoules S, Marler P. 1985 External reference and affective signaling. In The development of expressive behavior: biology-environment interactions (ed. G Zivin), pp. 77–101. Orlando, FL: Academic Press.

[24] Marler P. 1980 Primate vocalization: affective or symbolic? In Speaking of apes: a critical anthology of two-way communication with man, pp. 221–229. Boston, MA: Springer. (10.1007/978-1-4613-3012-7_13)

[25] Lima CF, Anikin A, Monteiro AC, Scott SK, Castro SL. 2019 Automaticity in the recognition of nonverbal emotional vocalizations. Emotion **19**, 219–233. (10.1037/emo0000429) 29792444 PMC6383750

[26] Spreckelmeyer KN, Kutas M, Urbach T, Altenmüller E, Münte TF. 2009 Neural processing of vocal emotion and identity. Brain Cogn. **69**, 121–126. (10.1016/j.bandc.2008.06.003) 18644670 PMC2642974

[27] Pell MD, Kotz SA. 2011 On the time course of vocal emotion recognition. PLoS ONE **6**, e27256. (10.1371/journal.pone.0027256) 22087275 PMC3210149

[28] Bickerton D. 1990 Language and species. Chicago, IL: University of Chicago Press.

[29] Darwin C. 1872 The expression of the emotions in animals and man. New York, NY: Appleton & Co.

[30] Suzuki TN. 2018 Alarm calls evoke a visual search image of a predator in birds. Proc. Natl Acad. Sci. USA **115**, 1541–1545. (10.1073/pnas.1718884115) 29378940 PMC5816198

[31] Suzuki TN. 2020 Other species’ alarm calls evoke a predator-specific search image in birds. Curr. Biol. **30**, 2616–2620.(10.1016/j.cub.2020.04.062) 32413306

[32] Prüfer K *et al*. 2012 The bonobo genome compared with the chimpanzee and human genomes. Nature **486**, 527–531. (10.1038/nature11128) 22722832 PMC3498939

[33] Sato Y, Kano F, Morimura N, Tomonaga M, Hirata S. 2022 Chimpanzees (Pan troglodytes) exhibit gaze bias for snakes upon hearing alarm calls. J. Comp. Psychol. **136**, 44–53. (10.1037/com0000305) 34855426

[34] Hurlbert SH. 1984 Pseudoreplication and the design of ecological field experiments. Ecol. Monogr. **54**, 187–211. (10.2307/1942661)

[35] Waller BM, Warmelink L, Liebal K, Micheletta J, Slocombe KE. 2013 Pseudoreplication: a widespread problem in primate communication research. Anim. Behav. **86**, 483–488. (10.1016/j.anbehav.2013.05.038)

[36] Van Strien JW, Franken IHA, Huijding J. 2014 Testing the snake-detection hypothesis: larger early posterior negativity in humans to pictures of snakes than to pictures of other reptiles, spiders and slugs. Front. Hum. Neurosci. **8**, 691. (10.3389/fnhum.2014.00691) 25237303 PMC4154444

[37] Masataka N, Koda H, Atsumi T, Satoh M, Lipp OV. 2018 Preferential attentional engagement drives attentional bias to snakes in Japanese macaques (Macaca fuscata) and humans (Homo sapiens). Sci. Rep. **8**, 17773. (10.1038/s41598-018-36108-6) 30538271 PMC6289998

[38] Isbell LA. 2006 Snakes as agents of evolutionary change in primate brains. J. Hum. Evol. **51**, 1–35. (10.1016/j.jhevol.2005.12.012) 16545427

[39] Bar M. 2003 A cortical mechanism for triggering top-down facilitation in visual object recognition. J. Cogn. Neurosci. **15**, 600–609. (10.1162/089892903321662976) 12803970

[40] Clarke E, Reichard UH, Zuberbühler K. 2015 Context-specific close-range ‘hoo’ calls in wild gibbons (Hylobates lar). BMC Evol. Biol. **15**, 56. (10.1186/s12862-015-0332-2) 25888361 PMC4389582

[41] Berthet M, Neumann C, Mesbahi G, Cäsar C, Zuberbühler K. 2018 Contextual encoding in titi monkey alarm call sequences. Behav. Ecol. Sociobiol. **72**, 8. (10.1007/s00265-017-2424-z) PMC9527189 36203497

[42] Watson SK, Townsend SW, Range F. 2018 Wolf howls encode both sender- and context-specific information. Anim. Behav. **145**, 59–66. (10.1016/j.anbehav.2018.09.005)

[43] Yin S, McCowan B. 2004 Barking in domestic dogs: context specificity and individual identification. Anim. Behav. **68**, 343–355. (10.1016/j.anbehav.2003.07.016)

[44] Girard-Buttoz C, Surbeck M, Samuni L, Tkaczynski P, Boesch C, Fruth B, Wittig RM, Hohmann G, Crockford C. 2020 Information transfer efficiency differs in wild chimpanzees and bonobos, but not social cognition. Proc. R. Soc. B **287**, 20200523. (10.1098/rspb.2020.0523) PMC7329035 32576115

[45] Clay Z, Zuberbühler K. 2011 Bonobos extract meaning from call sequences. PLoS ONE **6**, e18786. (10.1371/journal.pone.0018786) 21556149 PMC3083404

[46] Savage-Rumbaugh ES. 1993 The emergence of language. In Tools, language and cognition in human evolution (eds KR Gibson, T Ingold), pp. 86–108. Cambridge, UK: Cambridge University Press.

[47] Rabinowitz A. 2016 Linguistic competency of bonobos (Pan paniscus) raised in a language-enriched environment. Master’s thesis, Iowa State University, Ames, IA, USA.

[48] Clay Z, Zuberbuhler K. 2009 Food-associated calling sequences in bonobos, Pan paniscus. Anim. Behav. **77**, 1387–1396. (10.1016/j.anbehav.2009.02.016)

[49] R Core Team. 2023 R: a language and environment for statistical computing. Vienna, Austria: R Foundation for Statistical Computing.

[50] Clay Z, Archbold J, Zuberbühler K. 2015 Functional flexibility in wild bonobo vocal behaviour. PeerJ **3**, e1124. (10.7717/peerj.1124) 26290789 PMC4540007

[51] Manser MB, Bell MB, Fletcher LB. 2001 The information that receivers extract from alarm calls in suricates. Proc. R. Soc. Lond. B **268**, 2485–2491. (10.1098/rspb.2001.1772) PMC1088904 11747568

[52] Marler P, Evans CS, Hauser MD. 1992 Animal signals: motivational, referential, or both? In Nonverbal vocal communication: comparative and developmental perspectives (eds H Papoušek, U Jürgens, M Papoušek), pp. 66–86. Cambridge, UK: Cambridge University Press.

[53] White FJ, Wood KD. 2007 Female feeding priority in bonobos, Pan paniscus, and the question of female dominance. Am. J. Primatol. **69**, 837–850. (10.1002/ajp.20387) 17358018

[54] Furuichi T. 2011 Female contributions to the peaceful nature of bonobo society. Evol. Anthropol. **20**, 131–142. (10.1002/evan.20308) 22038769

[55] Terrace HS. 1979 Nim: a chimpanzee who learned sign language. New York, NY: Columbia University Press.

[56] Krause MA, Beran MJ. 2020 Words matter: reflections on language projects with chimpanzees and their implications. Am. J. Primatol. **82**, e23187. (10.1002/ajp.23187) 32830339

[57] Tomasello M. 2017 What did we learn from the ape language studies? In Bonobos, pp. 95–104. Oxford, UK: Oxford University Press. (10.1093/oso/9780198728511.003.0007)

[58] Lahiff N, Clay Z, Epping A, Taglialatela JP, Townsend S, Slocombe KE. 2025 Supplementary material from: Conspecific alarm calls, but not food-associated calls, elicit affect-based and object-based mental representations in a bonobo (Pan paniscus). FigShare. (10.6084/m9.figshare.c.7690714) PMC11879614 40046665

